# OCD symptom dimensions and treatment: a new dimension?

**DOI:** 10.1192/j.eurpsy.2022.1656

**Published:** 2022-09-01

**Authors:** I. Soares Da Costa, R. Moreira

**Affiliations:** CHUSJ, Psychiatry, Porto, Portugal

**Keywords:** OCD, clusters, Treatment, symptom dimensions

## Abstract

**Introduction:**

Background: Obsessive-compulsive disorder (OCD) is a condition that includes distressing obsessions and repetitive compulsions. Usually presents with a wide range of symptons normally grouped into different clusters or dimensions. clinical impression and some empirical data suggest that certain groups of symptoms or clusters are more responsive to treatment than others, thus it can help clinicians to better guide initial treatment choices and management of individual patients

**Objectives:**

Objetictive: to describe the symptom dimensions in a clinical database that includes patients accompannied in an obssessive conpulsive disorder consultation in a tertiary hospital in Portugal and to point out some differences in treatment outcomes.

**Methods:**

We searched Pubmed and Cochrane Library database for english language articles.

**Results:**

To date it appears that pharmacotherapy and CBT are an effective treatment for the various OCD dimensions, although not all dimensions have been adequately studied or respond well to treatment. Knowing a specific symptom profile may have implications in treatment components that clinicians should be aware of.

**Conclusions:**

More research will be needed to determine the best tailor treatments to each patient’s profile and Modifications to treatment may be needed. Clinical implications and directions for future research are discussed.

**Disclosure:**

No significant relationships.

